# Quality of horse F(ab’)_2_ antitoxins and anti-rabies immunoglobulins: protein content and anticomplementary activity

**DOI:** 10.1186/s40409-018-0153-z

**Published:** 2018-06-18

**Authors:** Carla Cristina Squaiella-Baptistão, Fábio Carlos Magnoli, José Roberto Marcelino, Osvaldo Augusto Sant’Anna, Denise V. Tambourgi

**Affiliations:** 1 Laboratório de Imunoquímica, Instituto Butantan, Av. Vital Brazil, 1500, São Paulo, SP CEP 05503-900 Brazil; 2 Seção de Processamento de Plasmas Hiperimunes, Instituto Butantan, Av. Vital Brazil, 1500, São Paulo, SP CEP 05503-900 Brazil

**Keywords:** Heterologous immunoglobulin, F(ab’)_2_ fragment, Antitoxins, Anti-rabies, Protein profile, Complement system

## Abstract

**Background:**

Among other applications, immunotherapy is used for the post-exposure treatment and/or prophylaxis of important infectious diseases, such as botulism, diphtheria, tetanus and rabies. The effectiveness of serum therapy is widely proven, but improvements on the immunoglobulin purification process and on the quality control are necessary to reduce the amount of protein aggregates. These may trigger adverse reactions in patients by activating the complement system and inducing the generation of anaphylatoxins. Herein, we used immunochemical methods to predict the quality of horse F(ab’)_2_ anti-botulinum AB, anti-diphtheric, antitetanic and anti-rabies immunoglobulins, in terms of amount of proteins and protein aggregates.

**Methods:**

Samples were submitted to protein quantification, SDS-PAGE, Western blot analysis and molecular exclusion chromatography. The anticomplementary activity was determined in vitro by detecting the production of C5a/C5a desArg, the most potent anaphylatoxin. Data were analyzed by one-way ANOVA followed by Tukey’s post-test, and differences were considered statistically significant when *p* < 0.05.

**Results:**

Horse F(ab’)_2_ antitoxins and anti-rabies immunoglobulin preparations presented different amounts of protein. SDS-PAGE and Western blot analyses revealed the presence of protein aggregates, non-immunoglobulin contaminants and, unexpectedly, IgG whole molecules in the samples, indicating the non-complete digestion of immunoglobulins. The chromatographic profiles of antitoxins and anti-rabies immunoglobulins allowed to estimate the percentage of contaminants and aggregates in the samples. Although protein aggregates were present, the samples were not able to induce the generation of C5a/C5a desArg in vitro, indicating that they probably contain acceptable levels of aggregates.

**Conclusions:**

Anti-botulinum AB (bivalent), anti-diphtheric, antitetanic and anti-rabies horse F(ab’)_2_ immunoglobulins probably contain acceptable levels of aggregates, although other improvements on the preparations must be carried out. Protein profile analysis and in vitro anticomplementary activity of F(ab’)_2_ immunoglobulin preparations should be included as quality control steps, to ensure acceptable levels of aggregates, contaminants and whole IgG molecules on final products, reducing the chances of adverse reactions in patients.

## Background

Botulism, diphtheria, tetanus and rabies are severe infectious diseases caused by different agents, which have in common the recommendation of using immunotherapy as post-exposure treatment and/or prophylaxis [1, 2]. Immunotherapy consists of the use of specific antibodies to neutralize the main causes of these afflictions. In the cases of botulism, diphtheria and tetanus, the main objective of immunotherapy is to neutralize toxins, but also to opsonize the bacteria, promoting complement-dependent bacteriolysis. Regarding rabies, antibodies aim to neutralize viral particles, block their entry into uninfected cells, and also to promote antibody-directed cell-mediated cytotoxicity (ADCC) of infected cells by natural killer cells [3].

Botulism is an acute severe neuroparalytic disease caused by bacterial exotoxins produced by distinct strains of *Clostridium*, mainly *Clostridium botulinum*. Botulinum neurotoxins are metalloproteases that block peripheral motor and autonomic nerves, leading to death when respiratory muscles are affected. Seven different botulinum toxins, named from A to G serotypes, have been described according to their antigenic properties. Three clinical variants include (1) food-borne botulism due to the ingestion of products contaminated with botulinum toxin; (2) wound botulism due to wound contamination with *Clostridium botulinum* spores that germinate and lead to the release of botulinum toxin; and (3) infant botulism due to the ingestion of spores, mainly present in honey, and multiplication within the gastrointestinal tract. Treatment includes hospital intensive support for mechanical ventilation and neutralization of toxins by passive immunization [3–5].

Diphtheria is an acute bacterial respiratory and systemic disease caused by the diphtheria toxin (DT), produced by three species of *Corynebacterium*: *C. diphtheriae*, which most commonly causes the disease in humans, *C. ulcerans* and *C. pseudotuberculosis*. DT is the main virulence factor of these bacteria and contributes to the formation of a typical pseudomembrane in the nasopharynx of patients, causing respiratory symptoms, such as cough and dyspnea. In addition, the circulating toxins are internalized by different cells and cause severe systemic complications, including myocarditis and neuritis. Along with appropriate antimicrobial therapy, treatment with diphtheria antitoxin serum must be immediately initiated in order to neutralize circulating toxins and prevent their binding to tissues [6].

Tetanus is a potentially fatal neurological disease caused by the tetanus neurotoxin, a protein produced by *Clostridium tetani*, an anaerobic bacterium whose resistant spores are commonly found in soil, feces and dirty objects. Wound contamination with tetanus spores lead them to germinate, allowing bacterial multiplication and toxin release. Tetanus toxin is internalized by the motor neurons at the neuromuscular junction, causing painful uncontrolled muscle contractions and increased sensitivity to audiovisual stimuli. The disease can be prevented by vaccination, but in rural areas of developing countries, where immunization sometimes is not available and where deliveries take place at home without adequate sterile procedures and in unclean environment, maternal and neonatal tetanus are very common. Contamination is usually via the umbilical stump. Treatment includes hospital care in an environment with reduced audiovisual stimuli, sedatives and muscle relaxants, in addition to tetanus antitoxin serum for neutralizing circulating toxins [1, 4, 7].

Rabies is a neurological disease caused by the rabies virus (RABV), which is a single-stranded, negative-sense RNA virus from *Lyssavirus* genus, *Rhabdoviridae* family. The virus is present in the saliva of infected animals, such as dogs and bats, and transmitted by animal bites to the human tissues at the bite site. After an incubation period, RABV spreads into the central nervous system, causing neuronal dysfunction, which leads to cardiorespiratory complications and multisystem organ failure. Once clinical symptoms develop, rabies is virtually always fatal. However, if post-exposure prophylaxis (PEP) is early carried out, virus dissemination can be controlled. PEP consists of vaccination combined with rabies immunoglobulin (RIG) administration, in addition to wound cleaning. RIG administration is recommended as soon as possible and not exceeding seven days after exposure to the virus. In several countries, human RIG is available, prepared from the plasma of immunized donors. In the absence of human, equine RIG can be used and shows similar clinical outcomes in preventing rabies [3, 8, 9].

In Brazil, anti-botulinum AB (bivalent), anti-diphtheric, antitetanic and anti-rabies heterologous immunoglobulins are all obtained from the plasma of immunized horses and consist of F(ab’)_2_ fragments obtained by pepsin digestion and ammonium sulphate precipitation. F(ab’)_2_ fragments are believed to cause less early adverse reactions than whole IgG. There are basically three types of early serum therapy reactions:

- IgE-mediated anaphylactic reactions, due to the presence of specific IgE in patients previously sensitized to any component present in the immunoglobulin preparation;
- non-IgE-mediated anaphylactic reactions, due to the activation of classical pathway of the complement system and generation of anaphylatoxins;
- pyrogenic reactions, due to the presence of endotoxin in the therapeutic preparations.

Non-IgE-mediated anaphylactic reactions constitute the majority of early reactions induced by therapeutic immunoglobulins. In theory, removal of the Fc portion of IgG could prevent these reactions [10]. However, various studies have shown that both IgG or F(ab’)_2_ heterologous immunoglobulins can activate the complement system in vitro [11, 12], and protein concentration and aggregation has been increasingly suggested to be the major cause of serum therapy early reactions [13–15].

Concerning the protein concentration, de Roodt et al. [16] showed that antivenoms with higher potency usually have higher amounts of protein *per* vial. The amount of protein contained in the final product is directly related to the eventual dose of extraneous protein to which patients will be exposed, increasing the chance of adverse reactions; thus, good preparations should contain low-concentration and high-affinity antibodies. To ensure high-quality products, the Brazilian National Health Surveillance Agency (ANVISA), concerned with the quality, safety and efficacy of new antitoxins, launched in 2017 the RDC 187, which indicates the performance of clinical trials for all new antitoxins or antivenoms that require registration for clinical use in Brazil [17]. According to this new guideline, the apilic antivenom that aims to treat massive Africanized honeybee stings is in clinical trial phase I/II for future registration by this regulatory agency [18].

Interestingly, the RDC 187 does not mention the necessity of testing the products concerning the presence of protein aggregates. Regarding this issue, our group recently showed that several samples of IgG and F(ab’)_2_ antivenoms activated the complement system in vitro [19]. In that work, we analyzed 32 batches of antivenoms from the Butantan (Brazil), Vital Brazil (Brazil) and Clodomiro Picado (Costa Rica) institutes produced between 2007 and 2010. Antivenoms from the Butantan and Vital Brazil institutes consisted of F(ab’)_2_ fragments obtained by pepsin digestion and ammonium sulphate precipitation, while those from the Clodomiro Picado Institute contained whole IgG purified by caprylic acid precipitation.

Antivenoms from the three institutes generated C3a (C3a/C3a desArg), but only the samples containing higher amounts of protein aggregates induced the production of C5a (C5a/C5a desArg), the most potent anaphylatoxin [19]. Thus, early reactions to serum therapy may be related to protein aggregate-mediated complement activation, instead of Fc-mediated complement activation. Besides, the production of C5a/C5a desArg could be used as a marker to predict the presence of protein aggregates, which could help the quality control process of heterologous immunoglobulin production. Therefore, protein content and profile of heterologous immunoglobulins, as well as their ability to induce the production of C5a/C5a desArg in vitro, could be analyzed by the manufacturers to ensure low concentration of protein aggregates.

Herein, we studied the protein content and profile of horse F(ab’)_2_ anti-botulinum AB, anti-diphtheric, antitetanic and anti-rabies immunoglobulins, as well as the production of C5a/C5a desArg in vitro, aiming to predict the product quality in terms of amount of proteins and protein aggregates.

## Methods

### Horse F(ab’)_2_ antitoxins and anti-rabies immunoglobulins

Commercial horse F(ab’)_2_ anti-botulinum AB (bivalent), anti-diphtheric, antitetanic and anti-rabies immunoglobulins were obtained from the Butantan Institute (São Paulo, SP, Brazil) (Table 1). Samples were maintained at 4 °C until use.

**Table 1:** Samples of F(ab’)_2_ antitoxins and anti-rabies immunoglobulins

Product	Batch	Fabrication	Additional Information
Anti-botulinum AB	0908161	07/2009	1 mL contains 375 IU of anti-type A and 275 IU of anti-type B antibodies
Anti-diphtheric	0812211/A	11/2008	1 mL contains 1000 IU of specific antibodies
Antitetanic	0907138/B	06/2009	1 mL contains 1000 IU of specific antibodies
Anti-rabies	0906112/C	04/2009	1 mL contains 200 IU of specific antibodies

### Protein concentration of horse F(ab’)_2_ antitoxins and anti-rabies immunoglobulins

The protein concentration of the samples was determined using the BCA method (Pierce BCA Protein Assay kit, USA), according to the manufacturer’s instructions, using bovine serum albumin (BSA – Sigma, USA) as standard.

### Polyacrylamide gel electrophoresis and Western blots of horse F(ab’)_2_ antitoxins and anti-rabies immunoglobulins

To determine protein profiles, samples were subjected to SDS-PAGE and Western blot analysis under non-reducing and reducing conditions. Briefly, immunoglobulin samples were diluted in saline solution (0.9% sodium chloride) to achieve the protein concentration of 2 mg/mL. Ten microliters of each diluted sample (20 μg of protein) was then mixed with the same volume of reducing or non-reducing buffer and subjected to 12% polyacrylamide gel electrophoresis in the presence of sodium dodecyl sulphate [20]. Molecular mass standards (Invitrogen, USA) were included in all runs, which were performed at 100 V. Gels were stained with silver [21]. For Western blot assays [22], proteins on unstained gels were transferred to nitrocellulose membranes at 150 mA. After the transfer, the membranes were blocked with 5% BSA in phosphate buffered saline (PBS –8.1 mM sodium phosphate, 1.5 mM potassium phosphate, 137 mM sodium chloride and 2.7 potassium chloride, p.H. 7.2) and then incubated with rabbit anti-horse IgG labelled with alkaline phosphatase (Sigma) diluted 1:7500. Nitroblue tetrazolium (NBT – Promega Corporation, USA) and 5-bromo-4-chloro-3-indolyl-phosphate (BCIP – Promega Corporation) were used to reveal the reactions, following the manufacturer’s recommendations.

### Chromatographic profiles of horse F(ab’)_2_ antitoxins and anti-rabies immunoglobulins

One milligram (1 mg) of commercial horse F(ab’)_2_ anti-botulinum AB (bivalent), anti-diphteric, antitetanic or anti-rabies immunoglobulins were subjected to molecular exclusion chromatography on a Superose 12 HR 10/30 column (Amersham Pharmacia Biotech AB, Sweden), equilibrated and eluted with ammonium acetate 50 mM, pH 7.4. Samples were run at a 24 mL/h flow rate, and their protein content was monitored by recording the absorbance at 280 nm in a UPC-900 Amersham Pharmacia Biotech.

### Normal human serum (NHS)

Human blood was obtained from adult healthy donors, aged between 25 and 35 years-old, men and women, who knew the objectives of the study and signed the corresponding informed consent form approved by the National Commission on Research Ethics – Research Ethics Committee of the Albert Einstein Hospital (CAAE02001612.6.0000.0071). Blood samples were collected without anticoagulant and allowed to clot for 4 h at 4 °C. After centrifugation, NHS was collected and stored at − 80 °C.

### Incubation of horse F(ab’)_2_ antitoxins and anti-rabies immunoglobulins with NHS

Samples were incubated with NHS, as a source of complement, for 1 h at 37 °C. The volume of NHS was the same for all incubations (200 μL), but the sample volume varied between the different immunoglobulins (Table 2), based on an estimation of the maximum volume of each immunoglobulin administered to patients, in proportion to the average volume of circulating plasma in a normal human adult. For practical purposes, a normal human adult was considered to have 2.75 L of circulating plasma (55% of 5 L of blood). For each control group, NHS was incubated with a corresponding volume of sterile non-pyrogenic saline (0.9% sodium chloride).

**Table 2:** Volume and amount of protein of each immunoglobulin incubated with normal human serum (NHS) in vitro

Product	Maximum volume administered to patients [volume (mL)/2.75 L of plasma]	Incubation volume^a^ [volume (μL)/200 μL of NHS]	Amount of protein (μg)/200 μL of NHS
Anti-botulinum AB	20	1.45	113.5 ± 0.71
Anti-diphteric	120	8.73	1409.0 ± 46.88
Antitetanic	20	1.45	118.8 ± 14.57
Anti-rabies	15	1.09	29.51 ± 1.05

^a^The incubation volume was based on the maximum volume administered to patients, proportionally to the volume of circulating plasma

### Detection of C5a/C5a desArg in NHS, after incubation with horse F(ab’)_2_ antitoxins and anti-rabies immunoglobulins

After incubating NHS with immunoglobulins or saline (control) as described above, the reactions were stopped by the addition of 10 mM of ethylene diamine tetracetic acid (EDTA – Sigma), and the concentration of C5a/C5adesArg was determined by ELISA (OptEIA ELISA kit – BD Biosciences, USA) following the manufacturer’s instructions.

### Statistical analysis

Data were analyzed by one-way ANOVA followed by Tukey’s post-test, and differences whose *p* values were less than 0.05 were considered to be statistically significant.

## Results

### Horse F(ab’)_2_ antitoxins and anti-rabies immunoglobulin preparations presented variable protein concentration

**Fig. 1:**
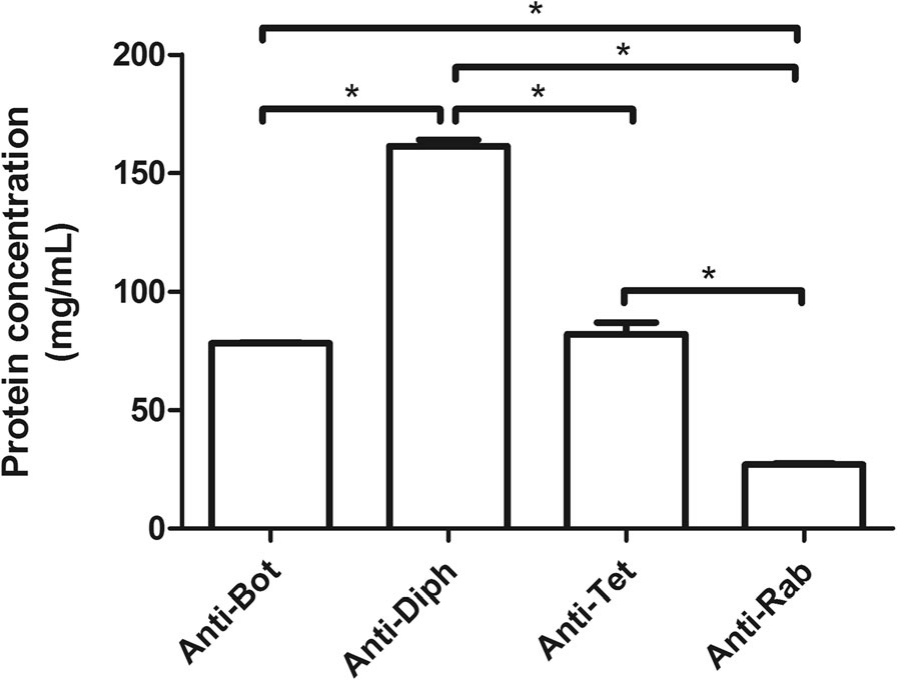
Protein concentration of horse F(ab’)_2_ antitoxins and anti-rabies immunoglobulins. The protein concentration of the samples was determined using the BCA method. Data represent the mean ± SD of two vials from the same batch for each serum type. *p < 0.05. Anti-Bot: Anti-botulinum AB; Anti-Diph: Anti-diphteric; Anti-Tet: Antitetanic; Anti-Rab: Anti-rabies

Protein detection by BCA method revealed that horse F(ab’)_2_ antitoxins and anti-rabies immunoglobulin preparations contained different amounts of heterologous proteins, with concentrations of approximately 27 mg/mL in anti-rabies sample, 80 mg/mL in anti-botulinum AB and antitetanic samples, and 160 mg/mL in anti-diphtheric sample (Fig. 1).

### Protein contaminants, aggregates and whole IgG molecules in horse F(ab’)_2_ antitoxins and anti-rabies immunoglobulins

**Fig. 2:**
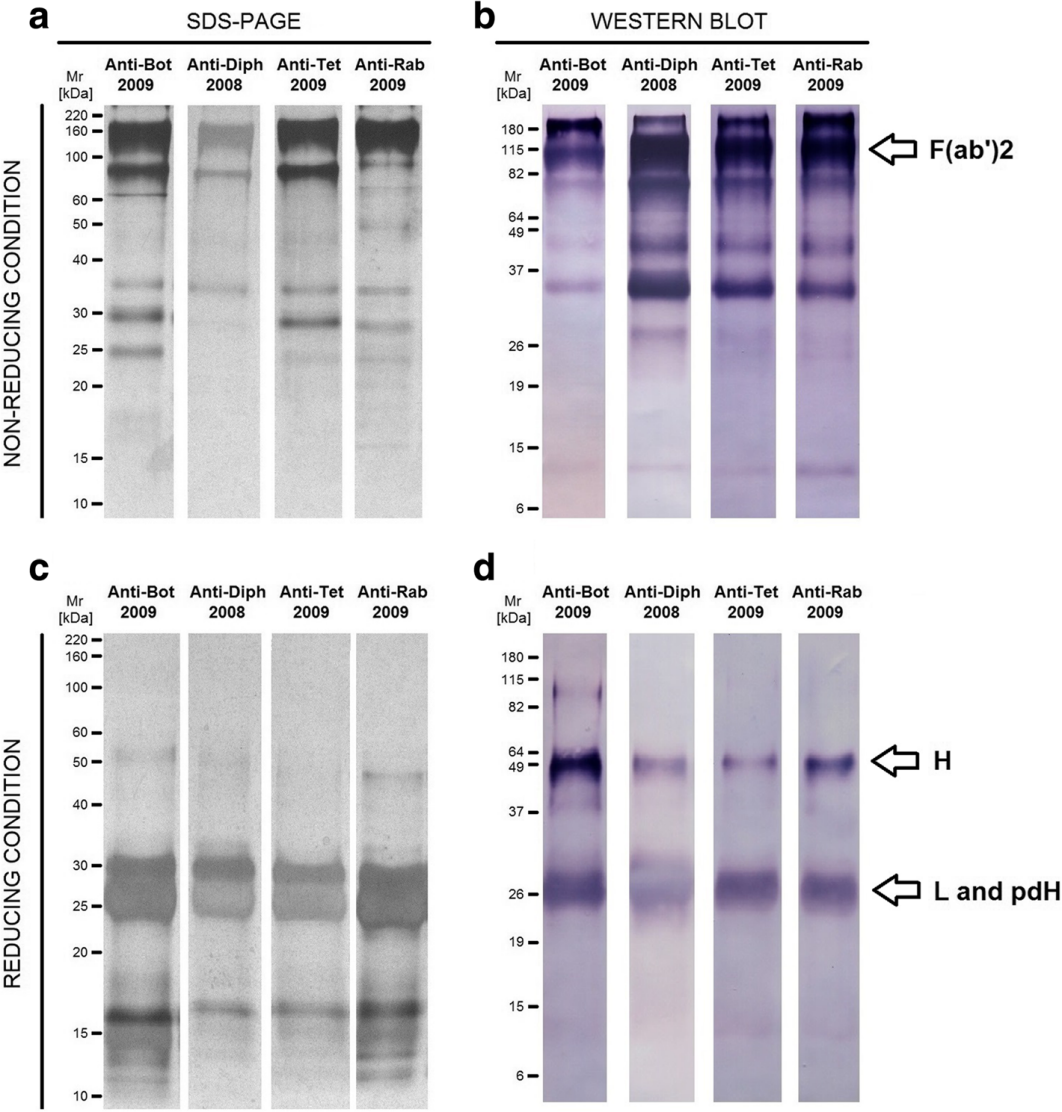
Polyacrylamide gel electrophoresis and Western blots of horse F(ab’)_2_ antitoxins and anti-rabies immunoglobulins. Serum samples were subjected to SDS-PAGE (**a** and **c**) and Western blot analysis (**b** and **d**) under non-reducing (**a** and **b**) and reducing (**c** and **d**) conditions. Molecular mass standards were included in all the runs and the relative molecular mass (Mr) are shown. Gels (**a** and **c**) were stained with silver and Western blot assays (**b** and **d**) were revealed with rabbit anti-horse IgG labelled with alkaline phosphatase. Anti-Bot: Anti-botulinum AB; Anti-Diph: Anti-diphteric; Anti-Tet: Antitetanic; Anti-Rab: Anti-rabies; H: heavy chain; L: light chain; pdH: pepsin-digested heavy chain

The protein profiles of antitoxins and anti-rabies immunoglobulins were determined by SDS-PAGE and Western blot. SDS-PAGE analysis, performed under non-reducing condition, showed the presence of several bands in all the samples, with molecular masses between 25 and 220 kDa (Fig. 2a), suggesting the presence of contaminants, aggregates and/or immunoglobulin fragmentation, since the expected molecular mass of F(ab’)_2_ fragments is ~ 110 kDa. The presence of non-immunoglobulin contaminants and of aggregates containing immunoglobulin fragments was confirmed by the Western blot reaction under non-reducing conditions. Figure 2b shows that the majority, but not all the bands observed in SDS-PAGE, were recognized by anti-horse IgG antibody.

Analysis, under reducing conditions, confirmed the presence of high molecular mass aggregates in the samples, which were disrupted by the reducing agent (Fig. 2c). The presence of non-immunoglobulin contaminants was also confirmed, since low molecular mass bands observed in reducing SDS-PAGE (Fig. 2c) were not detected by Western blot (Fig. 2d). As expected, analysis performed in reducing conditions revealed the presence of a ~ 25–30 kDa band in all the samples, corresponding to light and pepsin-digested heavy chains of IgG (Fig. 2d). However, unexpectedly, all the samples presented a ~ 50 kDa band, recognized by anti-horse IgG antibody (Fig. 2d), which corresponds to whole IgG heavy chain, indicating non-complete pepsin digestion of horse immunoglobulins.

### Molecular exclusion chromatography and quantification of contaminants in the immunoglobulin preparations

**Fig. 3:**
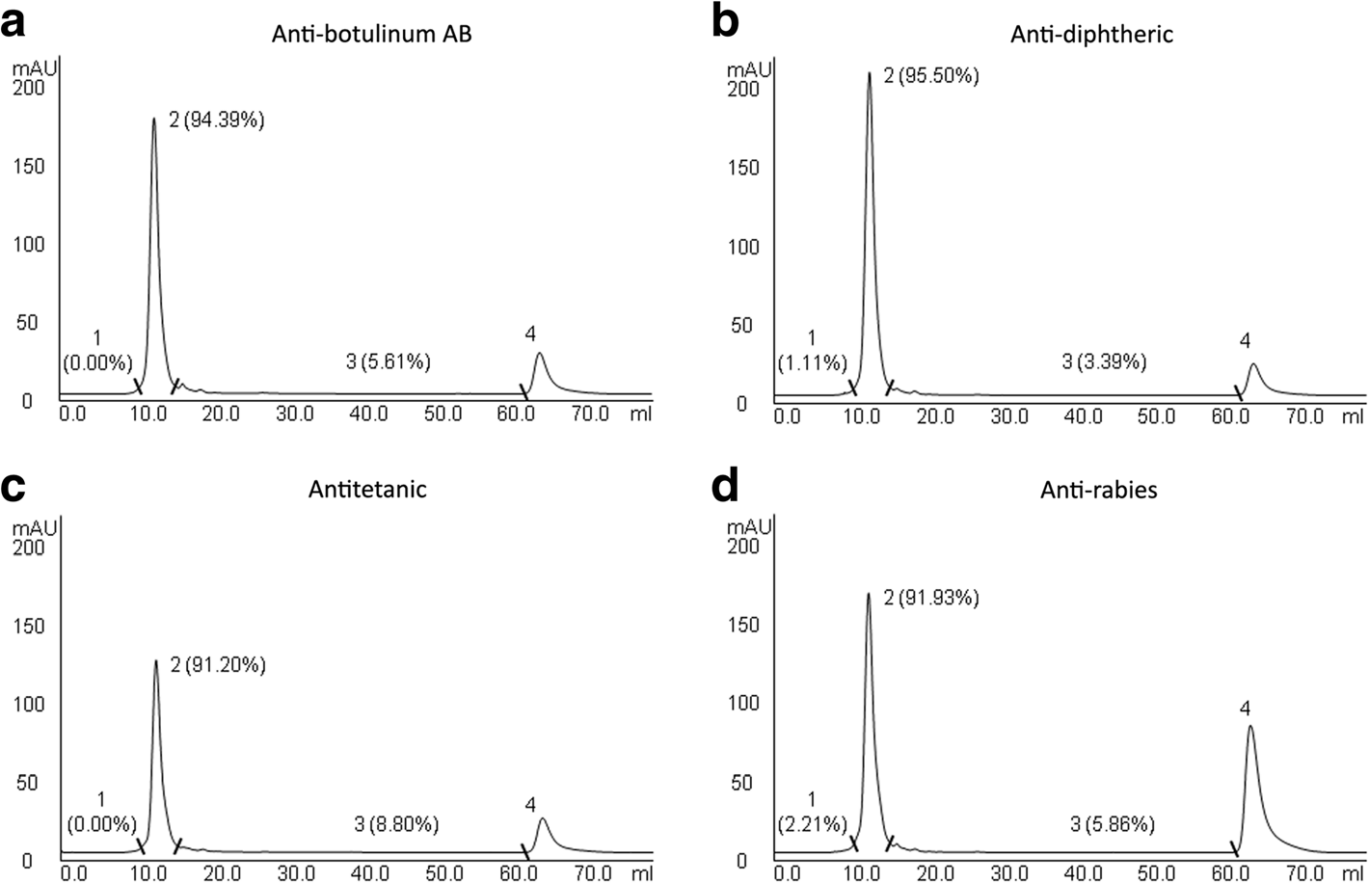
Chromatographic profiles of horse F(ab’)_2_ antitoxins and anti-rabies immunoglobulins. (**a**) Anti-botulinum AB, (**b**) anti-diphteric, (**c**) antitetanic and (**d**) anti-rabies sera were subjected to molecular exclusion chromatography on a Superose 12 HR 10/30 column at a 24 mL/h flow rate, and their protein content was monitored by recording the absorbance at 280 nm. The chromatograms were divided in four regions. The regions 1, 2 and 3 were considered for the calculation of the percentage of proteins in each region. The region 4 was considered to represent phenol used as preservative

Although not as sensible as SDS-PAGE and Western blot, to detect protein contaminants and aggregates, the chromatographic profiles of the samples of antitoxins and anti-rabies immunoglobulins allowed to estimate the percentage of contaminants and aggregates in the samples. In all chromatograms, it was observed similar profiles, that were divided in four regions:

- high molecular mass peaks (1), which include protein aggregates (Fig. 3);
- immunoglobulin peak (2), which includes non-digested IgG and F(ab’)_2_ fragments (Fig. 3);
- medium molecular mass peaks (3), which include contaminants (Fig. 3);
- low molecular mass peak (4), which probably represents phenol used as preservative, a small molecule that strongly absorbs at 280 nm, that was not considered for the calculation of protein content (Fig. 3).

Aggregates were not detected in anti-botulinum AB and antitetanic immunoglobulins by this analysis (Fig. 3a and c), while anti-diphtheric and anti-rabies immunoglobulins presented 1.11 and 2.21% of aggregates, respectively (Fig. 3b and d). Samples also presented variable amounts of contaminants, with approximately 5.6% of non-immunoglobulin proteins in anti-botulinum AB antitoxin (Fig. 3a), 3.4% in anti-diphtheric (Fig. 3b), 8.8% in antitetanic (Fig. 3c) and 5.9% in anti-rabies (Fig. 3d).

Anti-rabies preparation seemed to present high level of phenol (peak 4) compared to other samples (Fig. 3), but when the peak area was normalized by sample volume, the level was shown to be similar to other samples (data not shown) and within the reference limits [15].

### Horse F(ab’)_2_ antitoxins and anti-rabies immunoglobulins did not induce the generation of C5a/C5a desArg in vitro

**Fig. 4:**
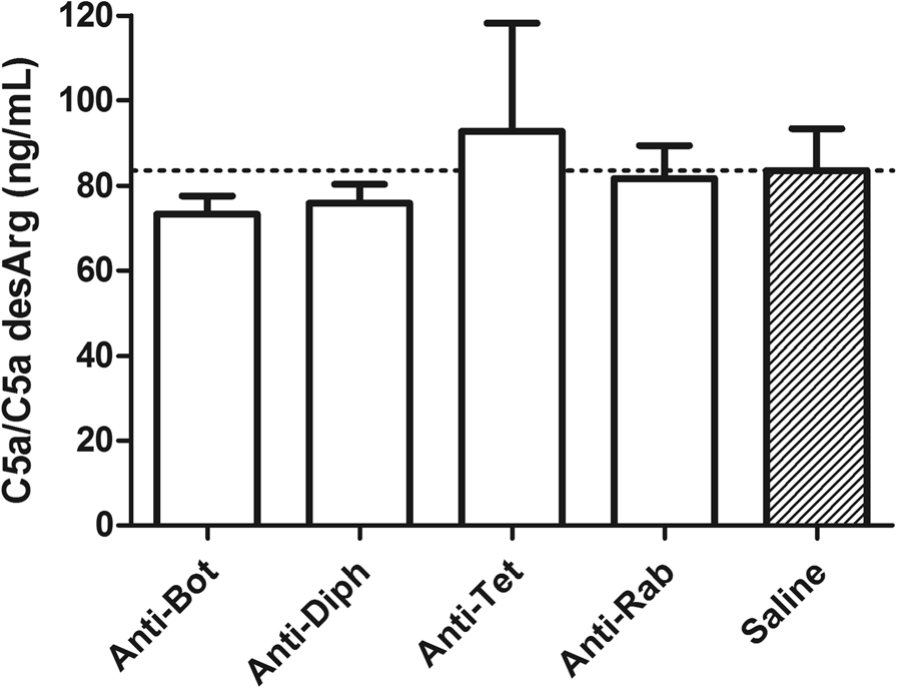
Detection of C5a/C5a desArg in NHS, after incubation with horse F(ab’)_2_ antitoxins and anti-rabies immunoglobulins. Samples were incubated with NHS or saline (control) according to the volumes shown in Table 2. The concentration of C5a/C5adesArg was determined by ELISA. Data represent the mean ± SD of two independent experiments using two vials from the same batch for each serum type. Anti-Bot: anti-botulinum AB; Anti-Diph: Anti-diphteric; Anti-Tet: Antitetanic; Anti-Rab: Anti-rabies

Antitoxins and anti-rabies immunoglobulins were incubated with NHS following the proportion in which these preparations would be used in patients, and the generation of C5a/C5a desArg was measured. In this condition, it was not detected the generation of C5a/C5a desArg (Fig. 4), independent of the amount of heterologous protein used in the experiment (Table 2).

## Discussion

Due to the severity and high mortality rate, botulism, diphtheria, tetanus and rabies are considered health emergencies and are all included as important health topics by the World Health Organization (WHO). In Brazil, these are notifiable diseases that are under the epidemiological surveillance of the Ministry of Health, and heterologous antitoxins and anti-rabies immunoglobulins are essential medicines produced, controlled and distributed by public manufacturers.

Herein, we analyzed the quality of these horse F(ab’)_2_ immunoglobulins, considering the protein content, electrophoretic profiles and the in vitro anticomplementary activity. Our previous data, analysing horse IgG and F(ab’)_2_ antivenoms, have shown the important role of protein aggregates to induce, in vitro, complement activation [19]. In such work, we demonstrated that antivenoms from the Butantan, Vital Brazil and Clodomiro Picado institutes generated C3a (C3a/C3a desArg), but only the samples containing higher amounts of protein aggregates induced the production of C5a (C5a/C5a desArg), the most potent anaphylatoxin [19]. The release of anaphylatoxins, mainly C5a, may promote the development of adverse reactions in patients. Therefore, the detection of protein aggregates, in antitoxins and anti-rabies immunoglobulins, could be useful to predict the quality of these immunoglobulin preparations. Moreover, other aspects of product quality, such as protein concentration and contaminants, were also analyzed.

Protein detection by BCA method revealed that horse F(ab’)_2_ antitoxins and anti-rabies immunoglobulin preparations contained different amounts of heterologous proteins, but only the anti-diphtheric immunoglobulin sample presented a protein concentration higher than 100 mg/mL, which is the upper limit recommended by the WHO [15]. However, this does not necessarily mean that this preparation is out of the range recommended by the Brazilian Ministry of Health, because the WHO allows the authorities of each country to establish their own limits [15]. Moreover, differences in the methods used to determine the protein concentration could explain the high protein concentration found by us. While quality control laboratories usually use the Biuret method to determine the protein concentration of samples, we used the BCA method, and the different chemical bases for protein detection in these two methods might explain some variation [23].

Coincidentally, besides being the most concentrated preparation, anti-diphtheric immunoglobulin is also the one used in the highest volume when administered to patients, reflecting in high amounts of heterologous protein. Administration of higher amounts of protein may be associated with higher adverse reactions rates, thus good preparations should contain low-concentration and high-affinity antibodies [15, 16]. However, it is not so easy to obtain such preparations, because of two factors: the intrinsic characteristics of the antigen that can interfere in its immunogenicity, and the variations in the immune response of individual horses that can result in antibodies with different affinities. These factors directly affect the quality of immunoglobulin preparations, and high protein concentration may be necessary to achieve the required neutralisation potency.

The diphtheria anatoxin, used as the immunization antigen for the production of horse F(ab’)_2_ anti-diphtheric immunoglobulin by the Butantan Institute, showed low immunogenicity in recent years, resulting in low potency preparations. A risk management plan for using low potency batches of anti-diphtheric immunoglobulin was prepared by the Brazilian Ministry of Health, which also authorized the extension of the expiration date, based on stability and potency control tests [24]. García et al. [25] showed that after a three-year storage period at 4 °C, antivenoms containing phenol or thimerosal as preservatives had an increased content of aggregates. Thus, the extension of expiration date could increase the chances of developing adverse reactions, therefore, tests for determining the amount of aggregates in immunoglobulin preparations should be adopted by quality control laboratories.

Herein, the protein aggregates in antitoxins and anti-rabies immunoglobulin preparations were relatively quantified by molecular exclusion chromatography. This technique showed the presence of 1.11% of aggregates in anti-diphtheric immunoglobulin and 2.21% of aggregates in anti-Rabies. These values are slightly lower than the observed by García et al. [25] using a similar methodology to determine the relative quantity of aggregates in IgG antivenoms, corroborating our previous data, in which we suggested the presence of higher amounts of protein aggregates in IgG rather than in F(ab’)_2_ preparations [19]. No aggregates were detected in anti-botulinum AB and antitetanic immunoglobulins by molecular exclusion chromatography, but high molecular bands were observed in SDS-PAGE and Western blot, showing these methods can be more sensible for this purpose.

Besides, SDS-PAGE and Western blot analysis also allowed to verify the presence of whole IgG molecules in some preparations, indicating the non-complete digestion of immunoglobulins by pepsin. This had already been observed for antivenoms produced by the Butantan Institute and indicates the necessity of improving the product quality, although does not seem to interfere with the in vitro anticomplementary activity [19].

Non-immunoglobulin proteins were also detected by SDS-PAGE and Western blot analyses, and the molecular exclusion chromatography was useful to relatively quantify these contaminants. High amounts of protein contaminants involve two problems: the patient is unnecessarily exposed to heterologous proteins, increasing the chances of adverse reactions; and there is an increase in the sample turbidity during storage, which is a signal of liquid instability [26].

Therefore, our analysis revealed the presence of aggregates, contaminants and non-digested immunoglobulins in the samples, but they did not induce the generation of C5a/C5a desArg in vitro. C5a is the most potent anaphylatoxin. It contains a C-terminal arginine residue that is rapidly cleaved by a serum carboxypeptidase, resulting in a desArg derivative. This mechanism is involved in the regulation of the complement system, but C5a desArg still exerts significant pro-inflammatory effects [27, 28].

Our previous data had already suggested a positive correlation between the in vitro generation of C5a/C5a desArg and the presence of protein aggregates in antivenoms [19]. Herein, although we detected aggregates in antitoxins and anti-rabies immunoglobulins, these samples did not induce the generation of C5a/C5a desArg, indicating they probably contain acceptable levels of aggregates.

## Conclusions

Protein profile analysis and in vitro anticomplementary activity of F(ab’)_2_ immunoglobulin preparations should be included as quality control steps to ensure acceptable levels of aggregates, contaminants and whole IgG molecules on final products, reducing the chances of adverse reactions in patients. Using the generation of C5a/C5a desArg in vitro as a marker for the presence of aggregates, anti-botulinum AB (bivalent), anti-diphtheric, antitetanic and anti-rabies horse F(ab’)_2_ immunoglobulins produced by the Butantan Institute comprise good quality products, probably inducing low rates of adverse reactions, although other improvements on the preparations should be carried out.

## Abbreviations

ADCC: Antibody-directed cell-mediated cytotoxicity
Anti-Bot: Anti-botulinum AB
Anti-Diph: Anti-diphteric
Anti-Rab: Anti-rabies
Anti-Tet: Antitetanic
ANVISA: Brazilian National Health Surveillance Agency
BCIP: 5-bromo-4-chloro-3-indolyl-phosphate
BSA: Bovine serum albumin
DT: Diphteria toxin
EDTA: Ethylene diamine tetracetic acid
NBT: Nitroblue tetrazolium
NHS: Normal human serum
PBS: Phosphate buffered saline
PEP: Post-exposure prophylaxis
RABV: Rabies virus
RIG: Rabies immunoglobulin
WHO: World Health Organization
